# Bacteria Isolated From the Antarctic Sponge *Iophon* sp. Reveals Mechanisms of Symbiosis in *Sporosarcina*, *Cellulophaga*, and *Nesterenkonia*

**DOI:** 10.3389/fmicb.2021.660779

**Published:** 2021-06-10

**Authors:** Mario Moreno-Pino, Juan A. Ugalde, Jorge H. Valdés, Susana Rodríguez-Marconi, Génesis Parada-Pozo, Nicole Trefault

**Affiliations:** ^1^GEMA Center for Genomics, Ecology and Environment, Faculty of Sciences, Universidad Mayor, Santiago, Chile; ^2^Millennium Initiative for Collaborative Research on Bacterial Resistance (MICROB-R), Santiago, Chile; ^3^Center for Genomics and Bioinformatics, Faculty of Sciences, Universidad Mayor, Santiago, Chile

**Keywords:** Antarctic sponges, symbiotic lifestyles, sponge microbiome, Antarctic ecosystem, sponge-associated bacteria

## Abstract

Antarctic sponges harbor a diverse range of microorganisms that perform unique metabolic functions for nutrient cycles. Understanding how microorganisms establish functional sponge–microbe interactions in the Antarctic marine ecosystem provides clues about the success of these ancient animals in this realm. Here, we use a culture-dependent approach and genome sequencing to investigate the molecular determinants that promote a dual lifestyle in three bacterial genera *Sporosarcina*, *Cellulophaga*, and *Nesterenkonia*. Phylogenomic analyses showed that four sponge-associated isolates represent putative novel bacterial species within the *Sporosarcina* and *Nesterenkonia* genera and that the fifth bacterial isolate corresponds to *Cellulophaga algicola*. We inferred that isolated sponge-associated bacteria inhabit similarly marine sponges and also seawater. Comparative genomics revealed that these sponge-associated bacteria are enriched in symbiotic lifestyle-related genes. Specific adaptations related to the cold Antarctic environment are features of the bacterial strains isolated here. Furthermore, we showed evidence that the vitamin B5 synthesis-related gene, *pan*E from *Nesterenkonia* E16_7 and E16_10, was laterally transferred within Actinobacteria members. Together, these findings indicate that the genomes of sponge-associated strains differ from other related genomes based on mechanisms that may contribute to the life in association with sponges and the extreme conditions of the Antarctic environment.

## Introduction

The sponge holobiont- or marine sponge-microorganism assemblage is considered one of the most basal and complex symbiotic relationships and proposed as one of the principal contributors to microbial diversity and functionality across global ocean ecosystems (Ruby, 2008; Pita et al., 2016; Thomas et al., 2016). The sponge holobiont is composed of a permanent, very dense, and diverse microbial community, dominated by the bacterial domain (Hentschel et al., 2006; Taylor et al., 2007). The sponge-associated bacteria participate actively in various fundamental metabolic processes including metabolizing sponge waste compounds, nutrient cycling (carbon, nitrogen, phosphorus, and sulfur), and production of secondary metabolites as chemical defensive molecules (Unson et al., 1994; Fan et al., 2012; Zhang et al., 2015).

Symbiotic functions are essential in the maintenance process of the host–microorganism relationship, which ultimately affects the health of the sponge holobiont. These functions, in turn, affect the health of the ecosystems (Pita et al., 2018). The establishment of the functional sponge–microbe interaction depends, in part, on a series of molecular determinants that mediate the symbiotic lifestyle adaptation (Gao et al., 2014; Tian et al., 2014, [Bibr B75][Bibr B9]; Zhang et al., 2019). These symbiotic signatures include eukaryotic-like protein domains (ELPs), clustered regularly interspaced short palindromic repeats (CRISPR), restriction-modification (R-M), and toxin–antitoxin (T-A) systems, which are involved in avoiding the digestion by the sponge host and defending against incoming foreign DNA (Magnuson, 2007; Nguyen et al., 2014; Horn et al., 2016; Díez-Vives et al., 2017; Slaby et al., 2017; Jahn et al., 2019). Fundamental insights into the specific strategies that promote the symbiotic lifestyle have been provided by genome studies from sponge symbionts (Slaby et al., 2017; Tian et al., 2017). The genome from *Candidatus Synechococcus spongiarum* revealed singular differences from free-living Cyanobacteria including the following: genome streamlining, lack of amino acid biosynthesis genes and the partial loss of DNA repair mechanisms, antioxidant enzymes, and photosynthetic complex genes (Gao et al., 2014; Burgsdorf et al., 2015). In the case of the sponge symbiont *Candidatus* Poribacteria, evidence of symbiotic lifestyle adaptation was inferred based on the loss of the flagellar structure, the presence of exclusive adhesion-related proteins, and a broad set of carbohydrate-degrading enzymes (Siegl et al., 2011; Zhang et al., 2019).

The study of sponge–microorganism symbiotic relationship is particularly relevant in Antarctica, where sponges are pivotal members of the benthic system with a high abundance and species-level endemism (McClintock et al., 2005; Downey et al., 2012). Antarctic sponge microbiomes are diverse, host specific, temporally stable, and different from tropical and temperate sponges (Webster et al., 2004; Rodríguez-Marconi et al., 2015; Savoca et al., 2019; Steinert et al., 2019). Functional insights of the Antarctic sponge-associated bacteria revealed that these bacteria produce antibacterial compounds that could control microbial populations inside the sponges (Papaleo et al., 2012). These compounds include a broad range of antibacterial compounds and xenobiotics (Steinert et al., 2019). More recently, the metagenomic analysis of two Antarctic sponge microbiomes showed the potential for nutrient cycling (carbon, nitrogen, sulfur, and phosphorus) and a high versatility of autotrophic carbon fixation (Moreno-Pino et al., 2020). Furthermore, enrichment of genes related to transposons, phages, CRISPR, T-A, and R-M systems was detected in these metagenomes, supporting the notion of genomic adaptations to symbiotic lifestyle in Antarctic sponges (Moreno-Pino et al., 2020).

Here, we describe molecular determinants of the Antarctic sponge microbiome interactions of five bacterial strains isolated from the Antarctic sponge *Iophon* sp. using a culture-dependent approach and genome sequencing. This study characterizes the mechanisms that promote a dual lifestyle in bacterial members belonging to the *Sporosarcina*, *Cellulophaga*, and *Nesterenkonia* genera. We aim to understand the genomic adaptations of these bacteria to live in association with the Antarctic sponges and whether their genomes present differences to the free-living counterparts.

## Materials and Methods

### Bacterial Cultures and Physiological Characterization

Seventeen bacterial strains were originally isolated from the Antarctic sponge *Iophon* sp. (class Demospongiae). The sponge sample was obtained in January 2014 and collected from Fildes Bay, King George Island, Antarctica (62° 12′ 11′′ S, 58° 55′ 15′′ W) at a depth of 5 m. For bacterial cultures and isolation, triplicate pieces (1 cm^3^) from the inner structure of the sponge tissue were cut with a sterile scalpel blade and were rinsed three times with sterilized seawater. The sponge tissues were homogenized, and the obtained extracts were plated in Marine Agar 2216 (BD-Difco) and incubated in the dark at 4°C for 30 days. Bacterial growth was examined weekly, and the bacterial colonies were isolated and transferred to Marine Broth 2216 (BD-Difco). Pure isolates were cryopreserved in Marine Broth supplemented with 10% glycerol at −80°C. The bacterial strains selected for genome sequencing included in this study were chosen based on their preliminary taxonomic assignation and the availability of reference genomes for comparative genomic analyses.

Strains E16_2, E16_3, E16_7, E16_8, and E16_10 were initially characterized by Gram staining, according to Dussault (1955). Enzymatic activities from these strains were tested with the API-ZYM Test System (bioMérieux) at 21°C for 3 days.

### Whole-Genome Sequencing and Bioinformatic Analysis

Genomic DNA was extracted using the GenElute bacterial genomic DNA kit (Sigma-Aldrich). DNA was used to prepare a sequencing library with NEBNext dsDNA Fragmentase kit (New England Biolabs, Ipswich, MA, United States) and quantified using a standard qPCR assay with a Library Quant Kit Illumina (Kapa). Libraries were sequenced using the Illumina Miseq platform with 2 × 150 cycles using the Miseq kit v.2. In order to improve sequence coverage of the strain E16_10, it was resequenced with a TruSeq Nano DNA LT Kit and using the Illumina HiSeq 4000 with 2 × 100 cycles. All raw whole-genome sequencing reads were filtered using PrinSeq (Schmieder and Edwards, 2011) (version 0.20.4). Adapters were removed with Cutadapt (version 1.10; -q 30) (Martin, 2011; Schmieder and Edwards, 2011). High-quality reads were assembled using SPAdes (version 3.10.1) with default parameters (Bankevich et al., 2012). Genome assembly of strain E16_10 was improved by performing genome scaffolding, combining the previously generated Miseq and Hiseq contigs with MeDuSa, using default parameters (Bosi et al., 2015). ORFs were predicted for all contigs >500 bp using Prodigal (version 2.6.3) (Hyatt et al., 2010). Functional annotation was done using eggNOG mapper v.1 against the eggNOG v.4.5 (Huerta-Cepas et al., 2017; see Table 1 for details).

**TABLE 1:** Summary of the genomic features of the Antarctic sponge-associated isolates.

	***Sporosarcina* sp. strain *E16_3***	***Sporosarcina* sp. strain *E16_8***	***Cellulophaga* sp. strain *E16_2***	***Nesterenkonia* sp. strain *E16_7***	***Nesterenkonia* sp. strain *E16_10***
Genome size	4,018,218	4,383,297	4,945,754	3,295,803	3,296,753
GC content (%)	40.8	40.7	33.5	67.3	67.3
No. of predicted genes	3,843	4,254	4,351	3,078	3,066
No. of CDS	3,790	4,178	4,308	3,025	3,014
No. of tRNA loci	40	61	36	47	46
No. of rRNA loci (5S)	5	6	1	1	1
No. of rRNA loci (16S)	2	3	1	1	1
No. of rRNA loci (23S)	1	1	1	1	1
No. ncRNAs	5	5	4	3	3
CRISPR Arrays	0	0	1	0	0
Completeness (%)	98.68	98.68	99.50	98.88	98.42
Contamination (%)	2.21	2.54	1.48	1.44	1.44
Heterogeneity (%)	0	0	0	0	0

Taxonomic classification of strains was performed using the full-length 16S rRNA gene sequences detected with barrnap version 0.7^1^, and RNAmmer 1.2 Server (Lagesen et al., 2007). Closely 16S rRNA gene sequences were found using BLASTn against NCBI-nr database (August 2019), with a cutoff of *E*-value < 10^–5^, in Geneious software version R10.2. The retrieved 10 best hits and sequences of neighboring representative lineages were aligned using MUSCLE (version 3.5) (Edgar, 2004), with 999 iterations, in Geneious version R10.2. The alignment was manually edited and used to construct Maximum Likelihood (ML) phylogenies. ML trees were generated using the best-fit models (K2 + GAMMA for *Cellulophaga* and *Sporosarcina*, and TN93+ GAMMA for *Nesterenkonia*) and were computed with 500 bootstrap replicates in MEGA7 (Kumar et al., 2016).

### Comparative Genomic Analyses

#### Fragment Recruitment

To estimate the abundance of genomes related to the bacterial strains E16_2, E16_3, E16_7, E16_8, and E16_10 in marine sponge microbiomes and planktonic communities, we performed a fragment recruitment analysis using 23 publicly available metagenomic datasets. These metagenomes included microbiomes associated with Antarctic sponges (*n* = 3), microbiomes from tropical and temperate sponges (*n* = 11), and marine microbial communities (*n* = 9), including one sample from surrounding seawater from the Antarctic sponges (see Supplementary Table 1 for details).

Metagenomic datasets were filtered based on a *Q* > 30 quality threshold using Prinseq (Schmieder and Edwards, 2011). Non-ribosomal reads were obtained using SortMeRNA (Kopylova et al., 2012), fragmented and filtered applying a previously published script (Plominsky et al., 2018) to obtain high-quality sequences within 50–100 bp. These were randomly subsampled according to the sample with the lowest number of reads across metagenomes (i.e., *Iophon* sp.). The recruitment of metagenomic sequences was performed using the genome of the bacterial strains from the Antarctic sponges as reference with FR-HIT (Niu et al., 2011) with a cutoff of 90% identity and 75% coverage. To further evaluate the relative abundance of genera associated with the five isolates obtained from Antarctic sponges, we performed a protein sequence classification of the metagenomes mentioned above. Non-ribosomal sequences were randomly subsampled according to the lowest reads across samples (*Iophon* sp.). The subsampled sequences were classified with Kaiju with default settings, aligning them against the NCBI BLAST nr +euk database (version 2019-06-25) (Menzel et al., 2016).

#### Pangenome Analysis

Pangenome analysis was performed by comparing 73 genomes from the *Sporosarcina*, *Cellulophaga*, and *Nesterenkonia* genera using the workflow implemented in the Anvi’o package^2^. Twenty-one percent of these genomes were complete, 16% were in the scaffold level, and 46% in the contig level (NCBI Sequence Read Archive version March 2019). We included 44 genomes from *Cellulophaga* spp., 17 genomes from *Sporosarcina* spp., and 12 genomes from *Nesterenkonia* spp. (see Supplementary Table 2 for details). Genomes were transformed to the Anvi’o format and filtered by contig length >500 bp (anvi-script-reformat-fasta; -l 500). Protein clustering was performed using BLAST and Markov Clustering (MCL) (van Dongen and Abreu-Goodger, 2012) algorithm with an inflation value of 0.1 (anvi-pan-genome; -minbit 0.5 –mcl-inflation 0.1 –use-ncbi-blast). Average nucleotide identity (ANI) was computed using BLAST (anvi-compute-ani; –method ANIb). Pangenomes were analyzed by clustering the genomes depending on whether the strains were host associated or free living.

Core and accessory genes obtained from the pangenome analysis were classified according to the following parameters. For core genes: maximum number of genes from each genome, 1; maximum functional homogeneity index, 0.9; minimum geometric homogeneity index, 1, minimum functional homogeneity index, 0.9; and maximum functional homogeneity index, 1. For accessory genes, only genes that were present in the host-associated strains were selected.

To test for significant differences in COG abundances between sponge-associated and free-living and between host-associated and free-living genomes, we used a two-tailed Wilcoxon rank-sum test in R environment using rstatix and STAMP 2.0.9 (Parks et al., 2014). COGs with a *p*-value < 0.05 were considered enriched. Analysis of similitude (ANOSIM) was used to test for significant differences between host-associated and free-living genomes using Primer 6 (PRIMER-E Ltd.).

#### Phylogenomics

The phylogenomic analysis was performed using the core genes. For this, 756, 355, and 190 core proteins for *Cellulophaga* spp., *Sporosarcina* spp., and *Nesterenkonia* spp., respectively, were concatenated and aligned using MUSCLE (version 3.5) (Edgar, 2004) with 999 iterations, and ambiguous positions were removed using trimAl v1.3 (-automated 1) (Capella-Gutiérrez et al., 2009). ML trees were performed using RAxML version 8 (Stamatakis, 2014), with 1,000 bootstrap replicates under the best-fit model for the three genera (LG + GAMMA + F). A multilocus sequence analysis (MLSA) based on 23 single-copy genes (SCG) shared between all genomes analyzed was performed to support the phylogenomic inferences (Supplementary Table 3; Wu et al., 2013). SCG were concatenated, aligned using MUSCLE (version 3.5) (Edgar, 2004) with 999 iterations in Geneious software version R10.2, and trimmed using trimAl v1.3 (Capella-Gutiérrez et al., 2009). ML trees were performed using RAxML (Stamatakis, 2014), with 1,000 bootstrap replicates, under the best-fit model (for *Nesterenkonia*, LG + GAMMA + F and for *Sporosarcina* and *Cellulophaga*, DAYHOFF + F).

### Horizontal Gene Transfer and Synteny Analyses

Genomic islands (GIs) were identified in the genomes of *Sporosarcina* sp. E16_3 and E16_8, *Cellulophaga* E16_2, and *Nesterenkonia* E16_7 and E16_10 using IslandViewer 4 with default settings (Bertelli et al., 2017). IslandViewer 4 integrates a comprehensive approach to predict GIs using four different methods: IslandPick, IslandPath-DIMOB, SIGI-HMM, and Islander.

The genomic GC content (%) from the GIs identified in *Nesterenkonia* E16_7 and E16_10 was computed with Geneious software version R10.2. Finally, the GI containing the ketopantoate reductase coding gene (*pan*E) was analyzed to confirm horizontal gene transfer (HGT) events using phylogenetic and comparative genomic analyses. The closest relatives of *pan*E gene were determined using bidirectional BLASTp against the NCBI-nr database (August 2019), retrieving the 50 non-redundant best hits. The *panE* genes were aligned using MUSCLE (version 3.5) with 999 iterations in Geneious software version R10.2, and ambiguous positions were removed using trimAl v1.3 (-automated 1) (Edgar, 2004; Capella-Gutiérrez et al., 2009). ML tree was performed using RAxML (Stamatakis, 2014), with 1,000 bootstrap replicates, under the LG + GAMMA + F model. Phylogenetic analysis of five neighboring genes to *panE* was also performed using 13--20 non-redundant best hits as described before. Additionally, to explore the synteny of *pan*E-related sequences in E16_7 and E16_10 strains, and *Nesterenkonia* sp. AN1 (NZ_JEMO00000000), and *Nesterenkonia aurantiaca* DSM27373 (NZ_SOAN00000000), contigs containing ORFs related to panE were extracted using Geneious version R10.2 and were compared with BLASTn using Trebol^3^.

## Results

**FIGURE 1:**
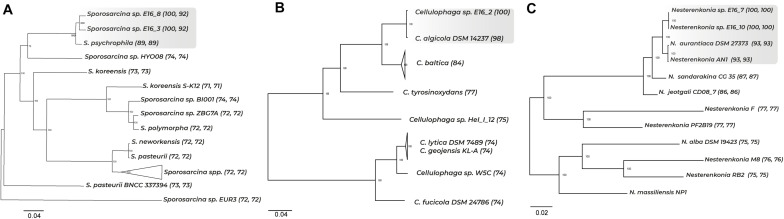
Phylogenomic tree of core genes and ANI among representative *Sporosarcina*, *Cellulophaga*, and *Nesterenkonia* genomes. A total of 756, 355, and 190 core genes were used to perform the phylogenomic tree *for Sporosarcina*, *Cellulophaga*, and *Nesterenkonia*. ANI values are shown in parenthesis. In **(A)**, first and second ANI values correspond to comparison with *Sporosarcina* E16_8 and E16_3, respectively; in **(B)**, ANI value corresponds to comparison with *Cellulophaga* E16_2, and in **(C)**, first and second ANI values correspond to comparison with *Nesterenkonia* E16_7 and E16_10, respectively.

Phylogenetic analyses using the full-length 16S rRNA gene sequences of the five bacterial strains isolated from the Antarctic sponge *Iophon* sp. showed that strains E16_3 and E16_8 form a strongly supported monophyletic group with *Sporosarcina globispora* and *Sporosarcina psychrophila* (Supplementary Figure 1A). A whole-genome comparison showed that these sponge-associated strains shared 92% of ANI between them and below 89% of ANI to all *Sporosarcina* genomes (Supplementary Figure 2A). Furthermore, core and SCG showed a separation between the genomes of *Sporosarcina* sp. E16_3 and E16_8, and *S. psychrophila*, suggesting a species divergence between them (Figure 1A and Supplementary Figure 3A).

For the strain E16_2, the 16S rRNA gene displayed a high similarity with *Cellulophaga algicola*, *Cellulophaga pacifica*, and *Cellulophaga baltica*, which formed a well-supported monophyletic clade (Supplementary Table 4 and Supplementary Figure 1B). *Cellulophaga* sp. E16_2 displayed ANI values from 74 to 98% among *Cellulophaga* genomes (Supplementary Figure 2B). The close relationship between *Cellulophaga* sp. E16_2 and *C. algicola* DSM 14237 was confirmed with SCG (Figure 1B and Supplementary Figure 3B). These results indicate a close phylogenetic relationship between *Cellulophaga* E16_2 and *C. algicola* DSM 14237.

The 16S rRNA gene of strains E16_7 and E16_10 showed a high similarity with *Nesterenkonia* sp. AN1, *Nesterenkonia aurantiaca*, and *Nesterenkonia lutea* (Supplementary Table 4). ML tree showed that the strains E16_7 and E16_10 cluster together with *Nesterenkonia* sp. AN1 and *N. aurantiaca* (Supplementary Figure 1C). *Nesterenkonia* E16_7 and E16_10 showed 100% of ANI between them, and below 93% when compared with all *Nesterenkonia* genomes (Supplementary Figure 2C). Phylogenomic trees showed a clear separation between the sponge-associated strains and Antarctic strains *Nesterenkonia* sp. AN1 and *N. aurantiaca* (Figure 1C and Supplementary Figure 3C).

Altogether, these results indicate that some strains isolated from the Antarctic sponge *Iophon* sp. correspond to new species within two different phyla, i.e., Firmicutes and Actinobacteria. *Sporosarcina* sp. E16_3 and E16_8 represent two putative novel species. *Nesterenkonia* sp. E16_7 and E16_10 correspond to a single putative novel species, while the fifth strain (E16_2) corresponds to *Cellulophaga algicola*, a member of the phylum Bacteroidetes.

A general enzymatic characterization of these five strains reflected their taxonomic separation. The strains share the presence of proteases (valine arylamidase, leucine arylamidase) and a phosphohydrolase. Phosphatases were unevenly distributed between the sponge-associated strains (Supplementary Figure 4 and Supplementary Table 5).

### Distribution of the Sponge-Associated Bacterial Genomes in Host-Associated Microbiomes and Seawater

**FIGURE 2:**
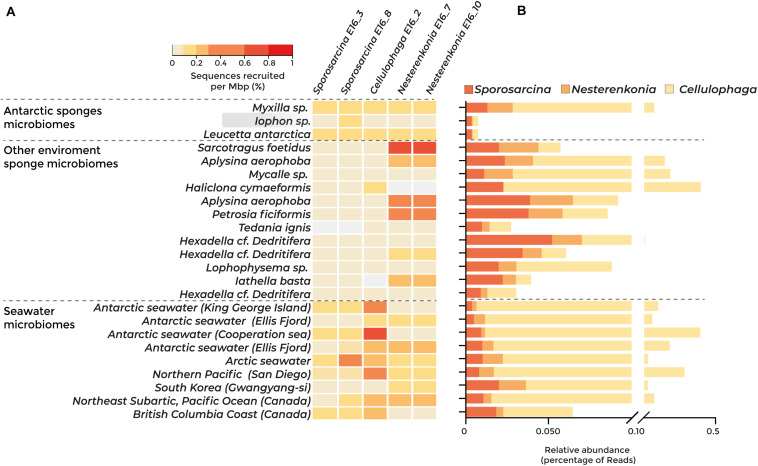
Abundance of the sponge-associated bacterial genomes among sponge and seawater microbiomes, determined by metagenomic fragment recruitment and taxonomic classification of protein sequences. Dashed lines separate Antarctic sponge microbiomes, sponge microbiomes from other environments, and seawater microbiomes. Highlighted in gray is the metagenome from the Antarctic sponge host of the isolated strains. **(A)** Heatmap of fragment recruitment analysis. The scale bar indicates the percentage of recruited sequences per megabase pair. **(B)** Abundance of sequences classified at the protein level belonging to *Sporosarcina*, *Cellulophaga*, and *Nesterenkonia* genera.

Fragment recruitment against 23 metagenomes showed that three of the five strains isolated have a higher abundance in seawater than in sponge microbiomes (Figure 2A). The relative abundance of *Sporosarcina* E16_3 and E16_8 was higher in the Antarctic sponge microbiomes *Myxilla* sp. and *Leucetta antarctica* and in seawater from Antarctica (Fildes Bay, King George Island, and Cooperation Sea), the Arctic, and the British Columbia coast. The genome of *Cellulophaga* E16_2 was most abundant in the Antarctic seawater and the Northern Pacific Ocean. However, we found the highest abundance of *Nesterenkonia* E16_10 and E16_7 in the non-Antarctic sponges *Sarcotragus foetidus*, *Aplysina aerophoba*, and *Petrosia ficiformis* (Figure 2A).

The taxonomic classification of the 23 metagenomic sequences revealed similar results, showing that *Sporosarcina*, *Nesterenkonia*, and *Cellulophaga* were in equal abundance in sponge microbiome and seawater samples (Figure 2B). Altogether, our results indicate that these sponge-associated bacteria inhabit marine sponges and their surrounding water, suggesting metabolic adaptations to thrive in both environments.

### Genes Related to the Symbiotic Lifestyle in the Antarctic Sponge Bacterial Strains

To explore the genomic adaptations that allow these bacteria to live in association with marine invertebrates in an extremely cold environment, we performed comparative genomic analyses focused on the genomes from host-associated bacteria. Summary of genomic features of sponge-associated strains is shown in Table 1. Among all genomes of *Sporosarcina* spp., *Cellulophaga* spp., and *Nesterenkonia* spp., we generated a total of 9,632, 3,312, and 4,483 protein clusters, and we predicted 2,595, 1,481, and 1,701 COGs, respectively.

**FIGURE 3:**
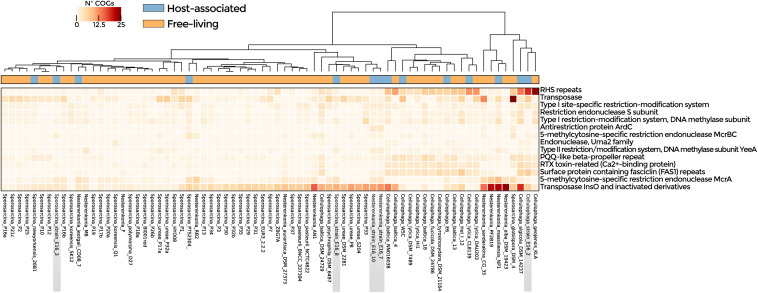
Symbiotic lifestyle functions detected in the genomes of *Nesterenkonia*, *Sporosarcina*, and *Cellulophaga*. COGs related to CRISPR, R-M, and T-A systems, transposases, and ELPs are shown. Heatmap represents the abundance of statistically significant COGs in host-associated and free-living genomes (*p*-value < 0.05).

Symbiosis-related genes were widespread among the analyzed genomes (Supplementary Table 6). ANOSIM according to lifestyle indicates that host-associated and free-living groups were similar although with some differences (R: 0.2). Enriched functions in host-associated genomes were T-A and R-M systems, transposases, and ELPs (*p*-value < 0.05) (Figure 3 and Supplementary Table 7), as well as transporter-encoding genes (osmotic response, efflux of threonine/homoserine lactone, and arsenic detoxification system) (Supplementary Figure 5). Genes involved in cold and osmotic stress response were widely distributed in the genomes analyzed (Supplementary Table 8). However, COGs related to Na+/H+ antiporter, universal stress protein family, and binding-protein-dependent transport system, were significantly enriched in the bacteria isolated from Antarctica in comparison with the strains isolated from other environments (Supplementary Figure 6 and Supplementary Table 9).

**FIGURE 4:**
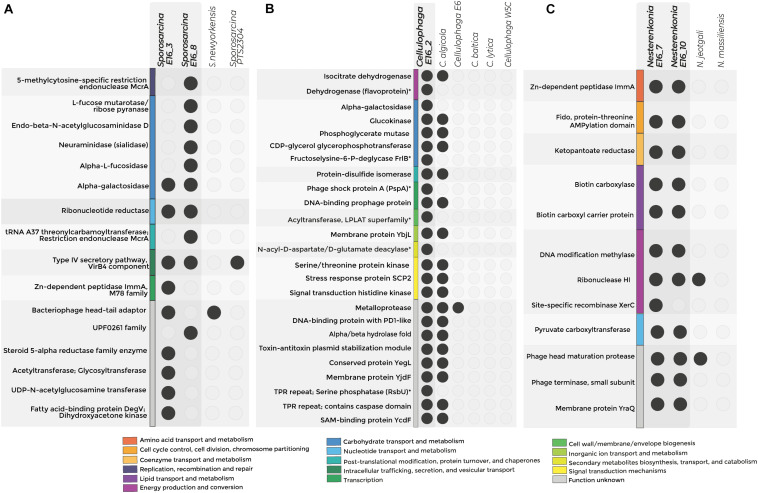
Exclusive COGs detected in the genome of host-associated bacteria. The figure shows the exclusive COG for *Sporosarcina* **(A)**, *Cellulophaga* **(B)**, and *Nesterenkonia* **(C)**. Colors represent categories of exclusive COGs, and empty circles denote the absence of the COG in the genome. *p-value < 0.05—functions without statistical support.

The genomes of *Sporosarcina* sp. PTS2304, *Sporosarcina* sp. P1, *Sporosarcina* sp. E16_8, *Nesterenkonia* E16_7, *Nesterenkonia* E16_10, *N. massiliensis*, and *N. jeotgali* CD08_7 were enriched in COGs related to transposases. The genomes of *Cellulophaga* sp. E16_2, *C. algicola* DSM14237, and *C. lytica* CL8139 in proteins with rearrangement hotspot RHS repeats, members of the ELPs. *Sporosarcina* E16_3 and E16_8 shared three COGs related to carbohydrate degradation, virulence, and tRNA modifications (Figure 4A and Supplementary Table 10).

Accessory COGs for *Sporosarcina* E16_3 were related to DNA recombination, degradation of sialic acids, and chitin. Specifically, accessory COGs related to neuraminidase and endo-β-*N*-acetylglucosaminidase suggest the potential for the digestion of structural polymers of sponge tissue (Figure 4A and Supplementary Tables 10,11).

For *Sporosarcina* E16_8, accessory COGs were related to cell wall biosynthesis and extracellular fatty acid incorporation into phospholipids. Specifically, UDP-*N*-acetylglucosamine enolpyruvyl transferase and the fatty acid kinase’s structural components, the fatty acid-binding protein, and dihydroxyacetone kinase were detected exclusively in *Sporosarcina* E16_8 (Figure 4A).

The Antarctic strains *Cellulophaga* E16_2 and *C. algicola* DSM 14237 shared COGs potentially involved in environmental adaptations to extremely cold conditions, e.g., correct formation of disulfide bonds for protein folding and tellurite resistance, as well as COGs linked to the symbiotic lifestyle, e.g., T-A system and protein-containing tetratricopeptide (TPR) repeats. From the seven accessory COGs that the genome from *Cellulophaga* sp. E16_2 contains, three were related to d-galactose degradation and environmental stressor response by the sigma factor (serine phosphatase RsbU) and phage shock protein (Psp) (Supplementary Table 10 and Figure 4B).

The pangenome analysis of the sponge-associated *Nesterenkonia* E16_7 and E16_10 revealed that their genomes shared COGs related to the T-A system (Zn-dependent peptidase ImmA), Mobile Genetic Elements (Phage head maturation protease and phage terminase), and pantothenate (vitamin B5) synthesis (Figure 4C and Supplementary Tables 10,11).

In all *Sporosarcina* genomes analyzed, the *pan*BCDE cluster is complete (Supplementary Table 12). This cluster codes for the vitamin B5 synthesis pathway, which is absent in sponges. *pan*BCDE cluster was complete in 26% of the available genomes of *Cellulophaga*. However, this cluster was exclusively found in the Antarctic sponge-associated *Nesterenkonia* strains (Supplementary Figure 7 and Supplementary Table 13).

Genes coding for the enzymes biotin carboxyl carrier protein of acetyl-CoA carboxylase (*pyc*A), biotin carboxylase subunit of acetyl coenzyme A carboxylase (*pyc*B), and pyruvate carboxylase (*pyc*C) involved in the central anaplerotic step in the TCA cycle were also detected exclusively in both sponge-associated *Nesterenkonia* strain (Figure 4C). Altogether, all the functions exclusively found in the Antarctic sponge-associated bacteria’s genomes could be related to genomic adaptations to cold environments and the host-associated lifestyle in the form of virulence factor as well as degradation of skeletal components of the sponges.

### Horizontal Gene Transfer Related to a Symbiotic Lifestyle

**FIGURE 5:**
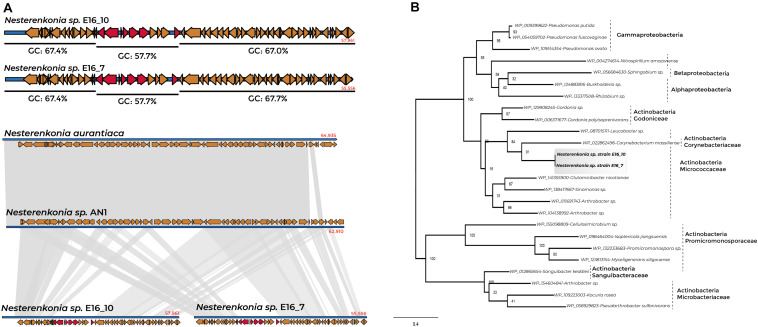
Synteny conservation and phylogenetic tree of *pan*E gene for sponge-associated *Nesterenkonia* strains and Actinobacteria members. **(A)** %GC content of the contig containing the *pan*E gene of *Nesterenkonia* E16_7 and E16_10 and genomic organization between sponge-associated *Nesterenkonia*, *N. aurantiaca*, and *Nesterenkonia* AN1. **(B)** Phylogenetic tree of *pan*E genes of *Nesterenkonia* E16_7 and E16_10 and Actinobacteria members.

We found 20 and nine GIs in the genomes of *Cellulophaga* E16_3 and E16_2, respectively, 20 GIs in *Sporosarcina* E16_8 and 34 in the sponge-associated *Nesterenkonia* strains (Supplementary Table 14). The presence of GIs indicated putative horizontal transfer events of the *pan*E gene in the genome of the sponge-associated *Nesterenkonia* strains. The genomic region containing the *pan*E gene represents a GI of 8,497 bp, characterized by a codon usage bias variation and a lower GC percentage than the GC-content for the whole genomic region (57.7% GC). This region is also characterized by a loose in the synteny in the genome region where the GI is present (Figure 5A).

Phylogenetic analysis of *pan*E-deduced protein from *Nesterenkonia* E16_7 and E16_10 revealed a relationship with *pan*E of the Actinobacteria *Corynebacterium massiliense* (member of the family Corynebacteriaceae), *Arthrobacter* sp., and *Glutamicibacter nicotianae* (members of the family Micrococcaceae) (Figure 5B and Supplementary Table 15). The adjacent genes to the *pan*E gene in the genomes of *Nesterenkonia* E16_7 and E16_10 show a close relationship with members belonging to the order Corynebacteriales (*Rhodococcus* sp., and *Corynebacterium* sp.), and Micrococcales (*Arthrobacter* sp., *Kocuria* sp., *Mycobacterium* sp., and *Micrococcus* sp.) (Supplementary Figure 8), indicating that most likely this complete gene cluster has undergone horizontal gene transfer within Actinobacteria members.

## Discussion

The sponge holobiont corresponds to one of the most complex symbiotic units, characterized by a huge microbial diversity that is considered higher than in planktonic communities globally (Thomas et al., 2016). This intimate host–microorganisms relationship is supported by a series of genomic adaptations to the symbiotic lifestyle that are still not fully understood. The whole-genome analysis presented here allows us to interrogate the genomic adaptations to the symbiotic lifestyle acquired by five Antarctic sponge-associated bacteria belonging to the genera *Cellulophaga*, *Sporosarcina*, and *Nesterenkonia*.

We conclude that sponge-associated *Sporosarcina* E16_3 and E16_8 and *Nesterenkonia* E16_7 and E16_10 may correspond to previously undescribed species. We follow multiple approaches to strengthen the analyzed isolates’ taxonomic classification, including ANI analysis and phylogenomics using core genes and SCG. Based on the conventional threshold of ≥95 ANI used for species delineation, the sponge-associated *Sporosarcina* and *Nesterenkonia* strains showed identity values below the species-level cutoff threshold (Konstantinidis et al., 2017). ANI is a widely accepted criterion for bacterial species classification purposes (Konstantinidis and Tiedje, 2005; Yarza et al., 2014; Konstantinidis et al., 2017; Jain et al., 2018) and has been helpful for the classification of sponge symbionts as *Ca.* Poribacteria (Kamke et al., 2013), *Ca. Synechococcus spongiarum* (Gao et al., 2014), *Pseudovibrio* sp. POLY-S9 (Alex and Antunes, 2015), and *Shewanella* sp. OPT22 (Alex and Antunes, 2019). Phylogenomic analysis further supports that sponge-associated *Sporosarcina* and *Nesterenkonia* are putative novel species. However, it is important to note that all genome-based taxonomy approaches are strongly affected by genome availability in public databases, which also applies to our study. Thus, efforts to increase whole-genome sequencing of sponge-associated bacteria would be relevant to expand our taxonomic observations described here.

Notably, we observe that the five bacterial strains isolated from the Antarctic sponge *Iophon* sp. grouped in a separated phylogenetic cluster consisting exclusively of Antarctic strains. The phylogenetic separation between Antarctic and non-Antarctic clades could be associated with environmental adaptation, as observed in several bacterial members, including *Arthrobacter*, *Pseudoalteromonas*, *Nesterenkonia*, and *Paenibacillus darwinianus* (Dsouza et al., 2014, 2015; Aliyu et al., 2016). Although the underlying molecular mechanisms involved in cold adaptation in bacteria are still not fully understood, these bacteria display specialized metabolism to survive under extremely cold conditions, like heat-shock proteins, cold-active enzymes, extracellular polymeric substances, and specialized metabolites production (Pearce, 2017; Caruso et al., 2018a,b; Millán-Aguiñaga et al., 2019).

Fragment recruitment indicated that the relative abundance of the Antarctic sponge-associated strains is relatively low in *Iophon* compared with other sponges and seawater. This could be due to a low intrinsic microbial abundance in their tissues or due to the technical impairments of cultivability of marine bacteria and sponge-associated bacteria (Hardoim et al., 2014; Esteves et al., 2016). A common categorization for sponge microbiomes is between high microbial abundance (HMA) and low microbial abundance (LMA) and refers to the abundance and diversity of bacterial cells inside the sponges (Hentschel et al., 2003). This dichotomy has profound implications for sponge physiology, as HMA sponges tend to have higher pumping rates and higher nutrient exchanges (Weisz et al., 2008; Ribes et al., 2012). The HMA and LMA dichotomy has not been thoroughly studied in the sponge species *Iophon* sp.; however, *Iophon methanophila* from asphalt seeps can be considered both LMA and HMA, due to its low diversity but high abundance of associated microorganisms (Rubin-Blum et al., 2019).

Here, we show that the bacterial strains characterized contain genes relevant to the host–microorganisms relationship under cold conditions. Between them, we detect the gene coding for fatty acid-binding protein and dihydroxyacetone kinase, in *Sporosarcina* E16_8. These enzymes participate in fatty acid modification of bacterial cell membranes which mediate the adaptation to growth in cold temperatures, as well described in *S. globispora* (Yoon et al., 2001; Diomandé et al., 2015). However, fatty acid synthesis is an energy-demanding process, their exogenous uptake and assimilation would be particularly relevant for optimizing cellular metabolic processes. The same was described in the gram-positive pathogen *Staphylococcus aureus*, which incorporates host-origin unsaturated fatty acids using the fatty acid kinase (Parsons et al., 2014). This process allows *S. aureus* to lead energetic metabolism to virulence factor production (Parsons et al., 2014).

Another example is the general stress response sigma factor B, which modulates the expression of antibiotic resistance and cold stress genes (Price et al., 2001). The factor sigma factor B requires the serine phosphatase, RsbU, to release the anti-sigma factor RsbW and respond to stressors (Pané-Farré et al., 2009). Our results show that RsbU is present exclusively in the genome of *Cellulophaga* E16_2, which makes us speculate that sigma factor B-regulator would be key to respond to cold-conditions as has been proposed previously in the genome of other Antarctic bacterial strains (Aliyu et al., 2016). However, we do not rule out that RsbU could also promote the establishment of the sponge–microorganism relationship based on their role in regulating antibiotic resistance genes (Price et al., 2001). Antibiotic production in the sponge holobiont is an important process in the control of microbial pathogens and their resistance has been described as an adaptive mechanism to survive in the sponge microbiome (Phelan et al., 2011; Mangano et al., 2013; Versluis et al., 2016; Almeida et al., 2019). Thus, we can suppose that sigma B-regulator can mediate antibiotic resistance in *Cellulophaga*, as has been proposed for the pathogenic bacteria *S. aureus* and *Listeria monocytogenes* (Price et al., 2001; Kumar et al., 2014). Future studies, including transcriptomic analysis, would help clarify the role these genes play in establishing the host–microorganism relationship under cold conditions.

Furthermore, we demonstrate that the genomes from the sponge-associated strains described here have molecular determinants related to their host-associated lifestyle. These determinants include ELPs, CRISPR, and the R-M and T-A systems, which are typically enriched and highly active in sponge symbiont genomes (Slaby et al., 2017). Another accessory function detected in the establishment of microorganisms in the sponge is the degradation of structural biopolymers of sponge tissue. It has been detected in diverse pathogens of invertebrates and also in the sponge-symbiont *Poribacteria* (Kamke et al., 2013; Podell et al., 2019). The demosponge skeleton is composed of polysaccharides, such as chitin, and mucopolysaccharides in their cell surface (Brunner et al., 2009; Ehrlich et al., 2018). Accordingly, we found that the genomes of the sponge-associated *Sporosarcina* species are enriched in chitinases and neuraminidases, supporting their sponge-associated lifestyle.

Finally, we show that sponge-associated *Nesterenkonia* possesses all the genes responsible for vitamin B5 synthesis. Pantothenate (vitamin B5) forms the core of coenzyme A and acyl carrier protein, being crucial in energy and fatty acid metabolism (Leonardi and Jackowski, 2007). In contrast to animals, some sponge–symbionts can perform *de novo* synthesis of B vitamins such as thiamine (vitamin B1), riboflavin (vitamin B2), pantothenate (vitamin B5), pyridoxine (vitamin B6), biotin (vitamin B7), and cobalamin (vitamin B12) to likely provide to the sponge their vitamin requirements (Engelberts et al., 2020). Moreover, our results strongly suggest that sponge-associated *Nesterenkonia* E16_7 and E16_10 acquired the gene encoding the ketopantoate reductase within Actinobacteria members by HGT. It has been demonstrated that HGT within sponge microbiomes plays a relevant role in the metabolic versatility of sponge-associated microorganisms (Gauthier et al., 2016). Thus, microorganisms within the Antarctic sponge likely perform *de novo* pantothenate synthesis as a genomic adaptation to inhabit the sponge holobiont.

## Conclusion

Our genomic analysis of five bacterial strains belonging to *Cellulophaga*, *Sporosarcina*, and *Nesterenkonia* reveals that their genomes contain unique genomic adaptations to life associated with the Antarctic sponges. The sponge-associated bacteria isolated differ with respect to their free-living counterparts by the presence of exclusive genes related to cold environment adaptation and host-associated lifestyle. These genomic features include fatty acids incorporation from host origin, antibiotic resistance, and degradation of sponge structural biopolymers. Furthermore, HGT in sponge-associated *Nesterenkonia* strains suggests a putative role of *pan*E gene in the symbiotic lifestyle in the cold Antarctic waters. Overall, genomic adaptations described in the five strains suggest a high metabolic versatility that may be involved in the capability to inhabit the sponge tissue and live in extremely cold seawater environments.

## Data Availability Statement

The datasets presented in this study can be found in online repositories. The names of the repository/repositories and accession number(s) can be found in the article/Supplementary Material.

## Author Contributions

MM-P and NT conceived the experiments and drafted the manuscript. MM-P, GP-P, and SR-M conducted the experiments. MM-P analyzed the data. MM-P, NT, JU, and JV interpreted the data. All authors reviewed and approved the final manuscript.

## Conflict of Interest

The authors declare that the research was conducted in the absence of any commercial or financial relationships that could be construed as a potential conflict of interest.

## Footnotes

1 http://www.vicbioinformatics.com/software.barrnap.shtml

2 http://merenlab.org/2016/11/08/pangenomics-v2

3 https://inf.imo-chile.cl/software/trebol.html

## References

[B1] Alex A. Antunes A. (2015). Whole genome sequencing of the symbiont *Pseudovibrio sp*. from the intertidal marine sponge *Polymastia penicillus* revealed a gene repertoire for host-switching permissive lifestyle. *Genome Biol. Evol.* 7 3022–3032. 10.1093/gbe/evv199 26519859 PMC5635592

[B2] Alex A. Antunes A. (2019). Whole-genome comparisons among the genus shewanella reveal the enrichment of genes encoding ankyrin-repeats containing proteins in sponge-associated bacteria. *Front. Microbiol.* 10:5. 10.3389/fmicb.2019.00005 30787909 PMC6372511

[B3] Aliyu H. De Maayer P. Cowan D. (2016). The genome of the Antarctic polyextremophile *Nesterenkonia* sp. AN1 reveals adaptive strategies for survival under multiple stress conditions. *FEMS Microbiol. Ecol.* 92:fiw032. 10.1093/femsec/fiw032 26884466

[B4] Almeida E. L. Rincón A. F. C. Jackson S. A. Dobson A. D. W. (2019). Comparative genomics of marine sponge-derived *Streptomyces* spp. isolates sm17 and sm18 with their closest terrestrial relatives provides novel insights into environmental niche adaptations and secondary metabolite biosynthesis potential. *Front. Microbiol.* 10:1713. 10.3389/fmicb.2019.01713 31404169 PMC6676996

[B5] Bankevich A. Nurk S. Antipov D. Gurevich A. A. Dvorkin M. Kulikov A. S. (2012). SPAdes: a new genome assembly algorithm and its applications to single-cell sequencing. *J. Comput. Biol.* 19 455–477. 10.1089/cmb.2012.0021 22506599 PMC3342519

[B6] Bertelli C. Laird M. R. Williams K. P. Simon Fraser University Research Computing Group, Lau B. Y. Hoad G. (2017). IslandViewer 4: expanded prediction of genomic islands for larger-scale datasets. *Nucleic Acids Res.* 45 W30–W35. 28472413 10.1093/nar/gkx343 PMC5570257

[B7] Bosi E. Donati B. Galardini M. Brunetti S. Sagot M.-F. Lió P. (2015). MeDuSa: a multi-draft based scaffolder. *Bioinformatics* 31 2443–2451. 10.1093/bioinformatics/btv171 25810435

[B8] Brunner E. Ehrlich H. Schupp P. Hedrich R. Hunoldt S. Kammer M. (2009). Chitin-based scaffolds are an integral part of the skeleton of the marine demosponge *Ianthella basta*. *J. Struct. Biol.* 168 539–547. 10.1016/j.jsb.2009.06.018 19567270 PMC2871032

[B9] Burgsdorf I. Slaby B. M. Handley K. M. Haber M. Blom J. Marshall C. W. (2015). Lifestyle evolution in cyanobacterial symbionts of sponges. *MBio* 6:e00391–15. 26037118 10.1128/mBio.00391-15 PMC4453008

[B10] Capella-Gutiérrez S. Silla-Martínez J. M. Gabaldón T. (2009). trimAl: a tool for automated alignment trimming in large-scale phylogenetic analyses. *Bioinformatics* 25 1972–1973. 10.1093/bioinformatics/btp348 19505945 PMC2712344

[B11] Caruso C. Rizzo C. Mangano S. Poli A. Di Donato P. Finore I. (2018a). Production and biotechnological potential of extracellular polymeric substances from sponge-associated antarctic bacteria. *Appl. Environ. Microbiol.* 84:e01624–17. 10.1128/AEM.01624-17 29180360 PMC5795064

[B12] Caruso C. Rizzo C. Mangano S. Poli A. Di Donato P. Nicolaus B. (2018b). Extracellular polymeric substances with metal adsorption capacity produced by *Pseudoalteromonas* sp. MER144 from Antarctic seawater. *Environ. Sci. Pollut. Res. Int.* 25 4667–4677. 10.1007/s11356-017-0851-z 29197057

[B13] Díez-Vives C. Moitinho-Silva L. Nielsen S. Reynolds D. Thomas T. (2017). Expression of eukaryotic-like protein in the microbiome of sponges. *Mol. Ecol.* 26 1432–1451. 10.1111/mec.14003 28036141

[B14] Diomandé S. E. Nguyen-The C. Guinebretière M.-H. Broussolle V. Brillard J. (2015). Role of fatty acids in *Bacillus* environmental adaptation. *Front. Microbiol.* 6:813. 10.3389/fmicb.2015.00813 26300876 PMC4525379

[B15] Downey R. V. Griffiths H. J. Linse K. Janussen D. (2012). Diversity and distribution patterns in high southern latitude sponges. *PLoS One* 7:e41672. 10.1371/journal.pone.0041672 22911840 PMC3404021

[B16] Dsouza M. Taylor M. W. Turner S. J. Aislabie J. (2014). Genome-based comparative analyses of antarctic and temperate species of *Paenibacillus*. *PLoS One* 9:e108009. 10.1371/journal.pone.0108009 25285990 PMC4186907

[B17] Dsouza M. Taylor M. W. Turner S. J. Aislabie J. (2015). Genomic and phenotypic insights into the ecology of Arthrobacter from Antarctic soils. *BMC Genomics* 16:36. 10.1186/s12864-015-1220-2 25649291 PMC4326396

[B18] Dussault H. P. (1955). An improved technique for staining red halophilic bacteria. *J. Bacteriol.* 70 484–485. 10.1128/jb.70.4.484-485.1955 13263323 PMC386254

[B19] Edgar R. C. (2004). MUSCLE: multiple sequence alignment with high accuracy and high throughput. *Nucleic Acids Res.* 32 1792–1797. 10.1093/nar/gkh340 15034147 PMC390337

[B20] Ehrlich H. Shaala L. A. Youssef D. T. A. Aksamitowska S. Ż Tsurkan M. Galli R. (2018). Discovery of chitin in skeletons of non-verongiid Red Sea demosponges. *PLoS One* 13:e0195803. 10.1371/journal.pone.0195803 29763421 PMC5953452

[B21] Engelberts J. P. Robbins S. J. de Goeij J. M. Aranda M. Bell S. C. Webster N. S. (2020). Characterization of a sponge microbiome using an integrative genome-centric approach. *ISME J.* 14 1100–1110. 10.1038/s41396-020-0591-9 31992859 PMC7174397

[B22] Esteves A. I. S. Amer N. Nguyen M. Thomas T. (2016). Sample processing impacts the viability and cultivability of the sponge microbiome. *Front. Microbiol.* 7:499. 10.3389/fmicb.2016.00499 27242673 PMC4876369

[B23] Fan L. Reynolds D. Liu M. Stark M. Kjelleberg S. Webster N. S. (2012). Functional equivalence and evolutionary convergence in complex communities of microbial sponge symbionts. *Proc. Natl. Acad. Sci. U.S.A.* 109 E1878–E1887. 22699508 10.1073/pnas.1203287109 PMC3390844

[B24] Gao Z.-M. Wang Y. Tian R.-M. Wong Y. H. Batang Z. B. Al-Suwailem A. M. (2014). Symbiotic adaptation drives genome streamlining of the cyanobacterial sponge symbiont “Candidatus *Synechococcus spongiarum*. *mbio* 5:e00079–14. 24692632 10.1128/mBio.00079-14 PMC3977351

[B25] Gauthier M.-E. A. Watson J. R. Degnan S. M. (2016). Draft genomes shed light on the dual bacterial symbiosis that dominates the microbiome of the coral reef sponge *Amphimedon queenslandica*. *Front. Mar. Sci.* 3:196. 10.3389/fmars.2016.00196

[B26] Hardoim C. C. P. Cardinale M. Cúcio A. C. B. Esteves A. I. S. Berg G. Xavier J. R. (2014). Effects of sample handling and cultivation bias on the specificity of bacterial communities in keratose marine sponges. *Front. Microbiol.* 5:611. 10.3389/fmicb.2014.00611 25477868 PMC4235377

[B27] Hentschel U. Fieseler L. Wehrl M. Gernert C. Steinert M. Hacker J. (2003). Microbial diversity of marine sponges. *Prog. Mol. Subcell. Biol.* 37 59–88. 10.1007/978-3-642-55519-0_3 15825640

[B28] Hentschel U. Usher K. M. Taylor M. W. (2006). Marine sponges as microbial fermenters. *FEMS Microbiol. Ecol.* 55 167–177. 10.1111/j.1574-6941.2005.00046.x 16420625

[B29] Horn H. Slaby B. M. Jahn M. T. Bayer K. Moitinho-Silva L. Förster F. (2016). An enrichment of CRISPR and other defense-related features in marine sponge-associated microbial metagenomes. *Front. Microbiol.* 7:1751. 10.3389/fmicb.2016.01751 27877161 PMC5099237

[B30] Huerta-Cepas J. Forslund K. Coelho L. P. Szklarczyk D. Jensen L. J. von Mering C. (2017). Fast genome-wide functional annotation through orthology assignment by eggNOG-Mapper. *Mol. Biol. Evol.* 34 2115–2122. 10.1093/molbev/msx148 28460117 PMC5850834

[B31] Hyatt D. Chen G.-L. Locascio P. F. Land M. L. Larimer F. W. Hauser L. J. (2010). Prodigal: prokaryotic gene recognition and translation initiation site identification. *BMC Bioinformatics* 11:119. 10.1186/1471-2105-11-119 20211023 PMC2848648

[B32] Jahn M. T. Arkhipova K. Markert S. M. Stigloher C. Lachnit T. Pita L. (2019). A phage protein aids bacterial symbionts in eukaryote immune evasion. *Cell Host Microbe* 26 542–550.e5. 31561965 10.1016/j.chom.2019.08.019

[B33] Jain C. Rodriguez-R L. M. Phillippy A. M. Konstantinidis K. T. Aluru S. (2018). High throughput ANI analysis of 90K prokaryotic genomes reveals clear species boundaries. *Nat. Commun.* 9:5114. 10.1038/s41467-018-07641-9 PMC6269478 30504855

[B34] Kamke J. Sczyrba A. Ivanova N. Schwientek P. Rinke C. Mavromatis K. (2013). Single-cell genomics reveals complex carbohydrate degradation patterns in poribacterial symbionts of marine sponges. *ISME J.* 7 2287–2300. 10.1038/ismej.2013.111 23842652 PMC3834845

[B36] Konstantinidis K. T. Rosselló-Móra R. Amann R. (2017). Uncultivated microbes in need of their own taxonomy. *ISME J.* 11 2399–2406. 10.1038/ismej.2017.113 28731467 PMC5649169

[B37] Konstantinidis K. T. Tiedje J. M. (2005). Genomic insights that advance the species definition for prokaryotes. *Proc. Natl. Acad. Sci. U.S.A.* 102 2567–2572. 10.1073/pnas.0409727102 15701695 PMC549018

[B38] Kopylova E. Noé L. Touzet H. (2012). SortMeRNA: fast and accurate filtering of ribosomal RNAs in metatranscriptomic data. *Bioinformatics* 28 3211–3217. 10.1093/bioinformatics/bts611 23071270

[B39] Kumar P. S. Kumar Y. N. Prasad U. V. Yeswanth S. Swarupa V. Vasu D. (2014). Comparative structural and functional analysis of staphylococcus aureus glucokinase with other bacterial glucokinases. *Indian J. Pharm. Sci.* 76 430–436. 25425757 PMC4243260

[B40] Kumar S. Stecher G. Tamura K. (2016). MEGA7: molecular evolutionary genetics analysis version 7.0 for bigger datasets. *Mol. Biol. Evol.* 33 1870–1874. 10.1093/molbev/msw054 27004904 PMC8210823

[B41] Lagesen K. Hallin P. Rødland E. A. Stærfeldt H.-H. Rognes T. Ussery D. W. (2007). RNAmmer: consistent and rapid annotation of ribosomal RNA genes. *Nucleic Acids Res.* 35 3100–3108. 10.1093/nar/gkm160 17452365 PMC1888812

[B42] Leonardi R. Jackowski S. (2007). Biosynthesis of pantothenic acid and Coenzyme A. *EcoSal Plus* 2:10. 10.1128/ecosalplus.3.6.3.4 26443589 PMC4950986

[B43] Magnuson R. D. (2007). Hypothetical functions of toxin-antitoxin systems. *J. Bacteriol.* 189 6089–6092. 10.1128/jb.00958-07 17616596 PMC1951896

[B44] Mangano S. Michaud L. Caruso C. Lo Giudice A. (2013). Metal and antibiotic resistance in psychrotrophic bacteria associated with the Antarctic sponge *Hemigellius pilosus* (Kirkpatrick, 1907). *Polar Biol.* 37 227–235. 10.1007/s00300-013-1426-1

[B45] Martin M. (2011). Cutadapt removes adapter sequences from high-throughput sequencing reads. *EMBnet J.* 17:10. 10.14806/ej.17.1.200

[B46] McClintock J. B. Amsler C. D. Baker B. J. van Soest R. W. M. (2005). Ecology of Antarctic marine sponges: an overview. *Integr. Comp. Biol.* 45 359–368. 10.1093/icb/45.2.359 21676781

[B47] Menzel P. Ng K. L. Krogh A. (2016). Fast and sensitive taxonomic classification for metagenomics with Kaiju. *Nat. Commun.* 7:11257. 10.1038/ncomms11257 PMC4833860 27071849

[B48] Millán-Aguiñaga N. Soldatou S. Brozio S. Munnoch J. T. Howe J. Hoskisson P. A. (2019). Awakening ancient polar Actinobacteria: diversity, evolution and specialized metabolite potential. *Microbiology* 165 1169–1180. 10.1099/mic.0.000845 31592756

[B49] Moreno-Pino M. Cristi A. Gillooly J. F. Trefault N. (2020). Characterizing the microbiomes of Antarctic sponges: a functional metagenomic approach. *Sci. Rep.* 10:645. 10.1038/s41598-020-57464-2 PMC6971038 31959785

[B50] Nguyen M. T. H. D. Liu M. Thomas T. (2014). Ankyrin-repeat proteins from sponge symbionts modulate amoebal phagocytosis. *Mol. Ecol.* 23 1635–1645. 10.1111/mec.12384 23980812

[B51] Niu B. Zhu Z. Fu L. Wu S. Li W. (2011). FR-HIT, a very fast program to recruit metagenomic reads to homologous reference genomes. *Bioinformatics* 27 1704–1705. 10.1093/bioinformatics/btr252 21505035 PMC3106194

[B52] Pané-Farré J. Jonas B. Hardwick S. W. Gronau K. Lewis R. J. Hecker M. (2009). Role of RsbU in controlling SigB activity in *Staphylococcus aureus* following alkaline stress. *J. Bacteriol.* 191 2561–2573. 10.1128/jb.01514-08 19201800 PMC2668408

[B53] Papaleo M. C. Fondi M. Maida I. Perrin E. Lo Giudice A. Michaud L. (2012). Sponge-associated microbial Antarctic communities exhibiting antimicrobial activity against *Burkholderia cepacia* complex bacteria. *Biotechnol. Adv.* 30 272–293. 10.1016/j.biotechadv.2011.06.011 21742025

[B54] Parks D. H. Tyson G. W. Hugenholtz P. Beiko R. G. (2014). STAMP: statistical analysis of taxonomic and functional profiles. *Bioinformatics* 30 3123–3124. 10.1093/bioinformatics/btu494 25061070 PMC4609014

[B55] Parsons J. B. Broussard T. C. Bose J. L. Rosch J. W. Jackson P. Subramanian C. (2014). Identification of a two-component fatty acid kinase responsible for host fatty acid incorporation by Staphylococcus aureus. *Proc. Natl. Acad. Sci. U.S.A.* 111 10532–10537. 10.1073/pnas.1408797111 25002480 PMC4115530

[B56] Pearce D. A. (2017). “Extremophiles in antarctica: life at low temperatures,” in *Adaption of Microbial Life to Environmental Extremes*, eds Stan-Lotter H. Fendrihan S. (Cham: Springer), 99–131. 10.1007/978-3-319-48327-6_5

[B57] Phelan R. W. Clarke C. Morrissey J. P. Dobson A. D. W. O’Gara F. Barbosa T. M. (2011). Tetracycline resistance-encoding plasmid from *Bacillus* sp. strain #24, isolated from the marine sponge *Haliclona simulans*. *Appl. Environ. Microbiol.* 77 327–329. 10.1128/aem.01239-10 21057017 PMC3019722

[B58] Pita L. Fraune S. Hentschel U. (2016). Emerging sponge models of animal-microbe symbioses. *Front. Microbiol.* 7:2102. 10.3389/fmicb.2016.02102 28066403 PMC5179597

[B59] Pita L. Rix L. Slaby B. M. Franke A. Hentschel U. (2018). The sponge holobiont in a changing ocean: from microbes to ecosystems. *Microbiome* 6 1–18. 29523192 10.1186/s40168-018-0428-1 PMC5845141

[B60] Plominsky A. M. Trefault N. Podell S. Blanton J. M. De la Iglesia R. Allen E. E. (2018). Metabolic potential and in situ transcriptomic profiles of previously uncharacterized key microbial groups involved in coupled carbon, nitrogen and sulfur cycling in anoxic marine zones. *Environ. Microbiol.* 20 2727–2742. 10.1111/1462-2920.14109 29575531

[B61] Podell S. Blanton J. M. Neu A. Agarwal V. Biggs J. S. Moore B. S. (2019). Pangenomic comparison of globally distributed poribacteria associated with sponge hosts and marine particles. *ISME J.* 13 468–481. 10.1038/s41396-018-0292-9 30291328 PMC6331548

[B62] Price C. W. Fawcett P. Cérémonie H. Su N. Murphy C. K. Youngman P. (2001). Genome-wide analysis of the general stress response in *Bacillus subtilis*. *Mol. Microbiol.* 41 757–774. 10.1046/j.1365-2958.2001.02534.x 11532142

[B63] Ribes M. Jiménez E. Yahel G. López-Sendino P. Diez B. Massana R. (2012). Functional convergence of microbes associated with temperate marine sponges. *Environ. Microbiol.* 14 1224–1239. 10.1111/j.1462-2920.2012.02701.x 22335606

[B64] Rodríguez-Marconi S. De la Iglesia R. Díez B. Fonseca C. A. Hajdu E. Trefault N. (2015). Characterization of bacterial, archaeal and eukaryote symbionts from antarctic sponges reveals a high diversity at a three-domain level and a particular signature for this ecosystem. *PLoS One* 10:e0138837. 10.1371/journal.pone.0138837 26421612 PMC4589366

[B65] Rubin-Blum M. Antony C. P. Sayavedra L. Martínez-Pérez C. Birgel D. Peckmann J. (2019). Fueled by methane: deep-sea sponges from asphalt seeps gain their nutrition from methane-oxidizing symbionts. *ISME J.* 13 1209–1225. 10.1038/s41396-019-0346-7 30647460 PMC6474228

[B66] Ruby E. G. (2008). Symbiotic conversations are revealed under genetic interrogation. *Nat. Rev. Microbiol.* 6 752–762. 10.1038/nrmicro1958 18794913 PMC3579588

[B67] Savoca S. Lo Giudice A. Papale M. Mangano S. Caruso C. Spanò N. (2019). Antarctic sponges from the Terra Nova Bay (Ross Sea) host a diversified bacterial community. *Sci. Rep.* 9:16135. 10.1038/s41598-019-52491-0 31695084 PMC6834628

[B68] Schmieder R. Edwards R. (2011). Quality control and preprocessing of metagenomic datasets. *Bioinformatics* 27 863–864. 10.1093/bioinformatics/btr026 21278185 PMC3051327

[B69] Siegl A. Kamke J. Hochmuth T. Piel J. Richter M. Liang C. (2011). Single-cell genomics reveals the lifestyle of Poribacteria, a candidate phylum symbiotically associated with marine sponges. *ISME J.* 5 61–70. 10.1038/ismej.2010.95 20613790 PMC3105677

[B70] Slaby B. M. Hackl T. Horn H. Bayer K. Hentschel U. (2017). Metagenomic binning of a marine sponge microbiome reveals unity in defense but metabolic specialization. *ISME J.* 11 2465–2478. 10.1038/ismej.2017.101 28696422 PMC5649159

[B71] Stamatakis A. (2014). RAxML version 8: a tool for phylogenetic analysis and post-analysis of large phylogenies. *Bioinformatics* 30 1312–1313. 10.1093/bioinformatics/btu033 24451623 PMC3998144

[B72] Steinert G. Wemheuer B. Janussen D. Erpenbeck D. Daniel R. Simon M. (2019). Prokaryotic diversity and community patterns in Antarctic continental shelf sponges. *Front. Mar. Sci.* 6:297. 10.3389/fmars.2019.00297

[B73] Taylor M. W. Radax R. Steger D. Wagner M. (2007). Sponge-associated microorganisms: evolution, ecology, and biotechnological potential. *Microbiol. Mol. Biol. Rev.* 71 295–347. 10.1128/mmbr.00040-06 17554047 PMC1899876

[B74] Thomas T. Moitinho-Silva L. Lurgi M. Björk J. R. Easson C. Astudillo-García C. (2016). Diversity, structure and convergent evolution of the global sponge microbiome. *Nat. Commun.* 7:11870. 10.1038/ncomms11870 PMC4912640 27306690

[B75] Tian R.-M. Sun J. Cai L. Zhang W.-P. Zhou G.-W. Qiu J.-W. (2016). The deep-sea glass sponge *Lophophysema eversa* harbours potential symbionts responsible for the nutrient conversions of carbon, nitrogen and sulfur. *Environ. Microbiol.* 18 2481–2494. 10.1111/1462-2920.13161 26637128

[B76] Tian R.-M. Wang Y. Bougouffa S. Gao Z.-M. Cai L. Zhang W.-P. (2014). Effect of copper treatment on the composition and function of the bacterial community in the sponge *Haliclona cymaeformis*. *MBio* 5:e01980. 10.1128/mBio.01980-14 PMC4222105 25370493

[B77] Tian R.-M. Zhang W. Cai L. Wong Y.-H. Ding W. Qian P.-Y. (2017). Genome reduction and microbe-host interactions drive adaptation of a sulfur-oxidizing bacterium associated with a cold seep sponge. *mSystems* 2:e00184–16. 10.1128/mSystems.00184-16 28345060 PMC5361782

[B78] Unson M. D. Holland N. D. Faulkner D. J. (1994). A brominated secondary metabolite synthesized by the cyanobacterial symbiont of a marine sponge and accumulation of the crystalline metabolite in the sponge tissue. *Mar. Biol.* 119 1–11. 10.1007/bf00350100

[B79] van Dongen S. Abreu-Goodger C. (2012). Using MCL to extract clusters from networks. *Methods Mol. Biol.* 804 281–295. 10.1007/978-1-61779-361-5_15 22144159

[B80] Versluis D. Rodriguez de Evgrafov M. Sommer M. O. A. Sipkema D. Smidt H. van Passel M. W. J. (2016). Sponge microbiota are a reservoir of functional antibiotic resistance genes. *Front. Microbiol* 7:1848. 10.3389/fmicb.2016.01848 27909433 PMC5112248

[B81] Webster N. S. Negri A. P. Munro M. M. H. G. Battershill C. N. (2004). Diverse microbial communities inhabit Antarctic sponges. *Environ. Microbiol.* 6 288–300. 10.1111/j.1462-2920.2004.00570.x 14871212

[B82] Weisz J. B. Lindquist N. Martens C. S. (2008). Do associated microbial abundances impact marine demosponge pumping rates and tissue densities? *Oecologia* 155 367–376. 10.1007/s00442-007-0910-0 18030495

[B83] Wu D. Jospin G. Eisen J. A. (2013). Systematic identification of gene families for use as “markers” for phylogenetic and phylogeny-driven ecological studies of bacteria and archaea and their major subgroups. *PLoS One* 8:e77033. 10.1371/journal.pone.0077033 24146954 PMC3798382

[B84] Yarza P. Yilmaz P. Pruesse E. Glöckner F. O. Ludwig W. Schleifer K.-H. (2014). Uniting the classification of cultured and uncultured bacteria and archaea using 16S rRNA gene sequences. *Nat. Rev. Microbiol.* 12 635–645. 10.1038/nrmicro3330 25118885

[B85] Yoon J. H. Lee K. C. Weiss N. Kho Y. H. Kang K. H. Park Y. H. (2001). *Sporosarcina aquimarina* sp. nov., a bacterium isolated from seawater in Korea, and transfer of *Bacillus globisporus* (Larkin and Stokes 1967), *Bacillus psychrophilus* (Nakamura 1984) and *Bacillus pasteurii* (Chester 1898) to the genus *Sporosarcina* as *Sporosarcina globispora* comb. nov., *Sporosarcina psychrophila* comb. nov. and *Sporosarcina pasteurii* comb. nov., and emended description of th. *Int. J. Syst. Evol. Microbiol.* 51 1079–1086. 10.1099/00207713-51-3-1079 11411676

[B86] Zhang F. Blasiak L. C. Karolin J. O. Powell R. J. Geddes C. D. Hill R. T. (2015). Phosphorus sequestration in the form of polyphosphate by microbial symbionts in marine sponges. *Proc. Natl. Acad. Sci. U.S.A.* 112 4381–4386. 10.1073/pnas.1423768112 25713351 PMC4394258

[B87] Zhang S. Song W. Wemheuer B. Reveillaud J. Webster N. Thomas T. (2019). Comparative genomics reveals ecological and evolutionary insights into sponge-associated thaumarchaeota. *mSystems* 4:e00288–19. 10.1128/msystems.00288-19 31409660 PMC6697440

