# Supplementary material for: Bacteria Isolated From the Antarctic Sponge Iophon sp. Reveals Mechanisms of Symbiosis in Sporosarcina, Cellulophaga, and Nesterenkonia
Source: Front Microbiol. 2021 Jun 10;12:660779. doi: 10.3389/fmicb.2021.660779 (PMC8222686; doi:10.3389/fmicb.2021.660779)
Supplement: Supplementary file 1 [file Data_Sheet_1.PDF]

Supplementary Material

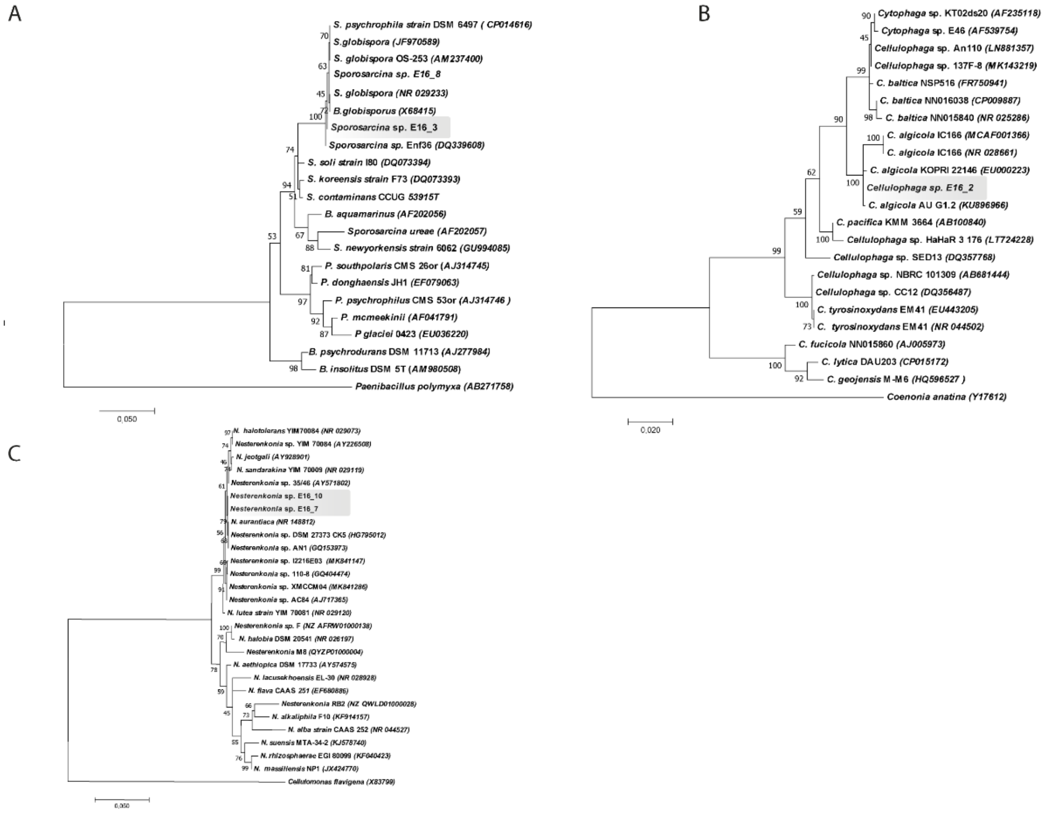

Supplementary Figure 1. Maximum Likelihood trees based on 16S rRNA gene sequences among representative *Sporosarcina*, *Cellulophaga*, and *Nesterenkonia*. The trees show the relationship between the sponge-associated bacterial isolated from the Antarctic sponge *Iophon* sp. and close taxa for *Sporosarcina* spp. **(A),** *Cellulophaga* spp. **(A),** and *Nesterenkonia* spp. **(C).** Bootstrap values (500 replications) are shown as percentage at each node.percentage at each node. The grey boxes highlight the strains from this study.

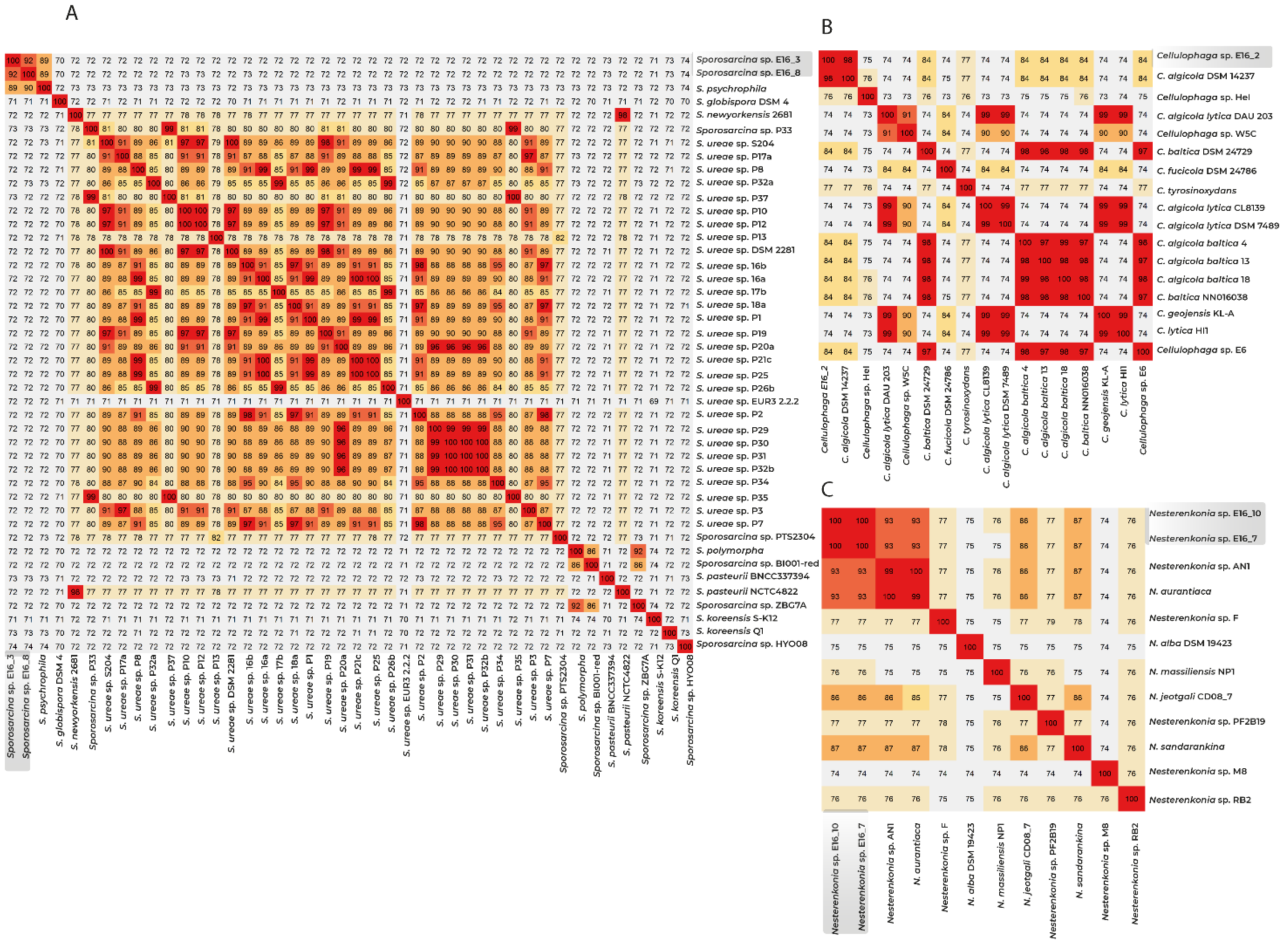

Supplementary Figure 2. Heat map of average nucleotide identity (ANI) among representative *Sporosarcina*, *Cellulophaga*, and *Nesterenkonia*. The percentage of ANI for *Sporosarcina* spp. **(A),** *Cellulophaga* spp. **(A),** and *Nesterenkonia* spp. **(C)** is represented. The grey boxes highlight the strains from this study.

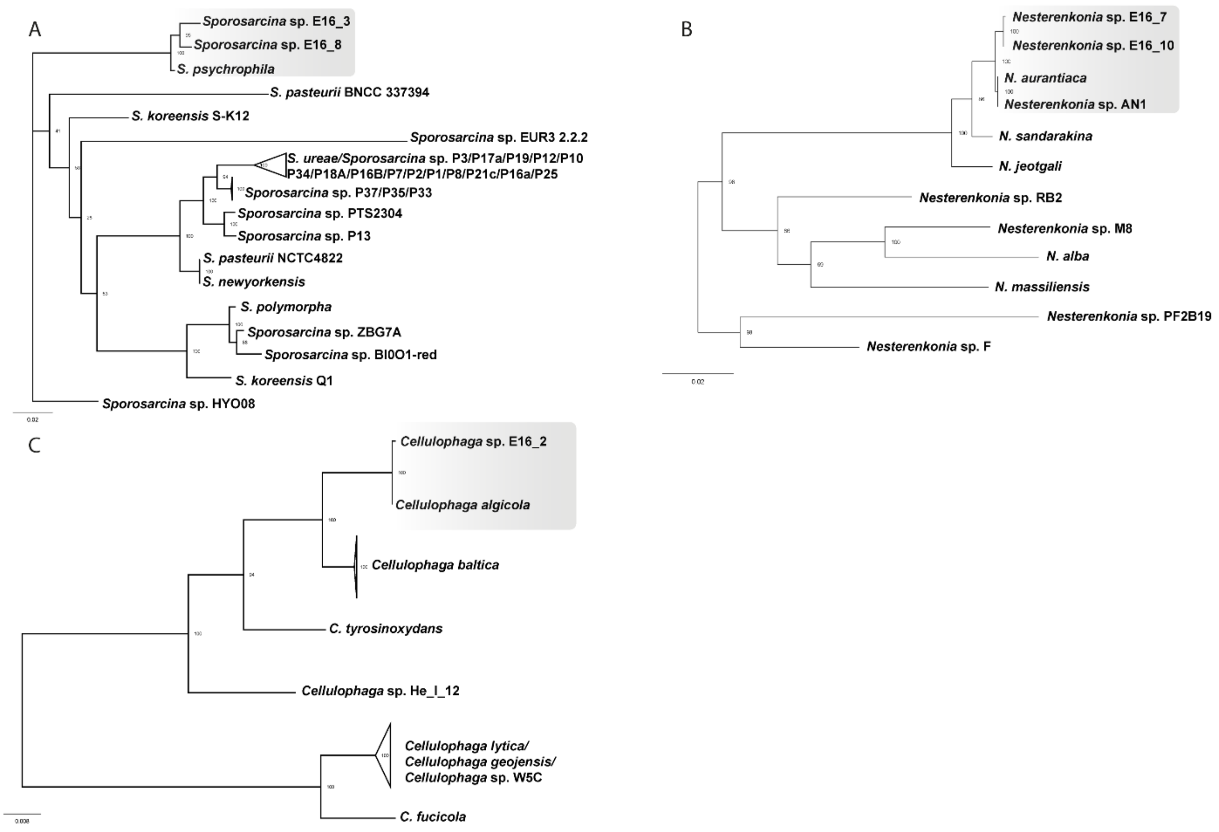

Supplementary Figure 3. Maximum Likelihood trees based on 23 SCG gene sequences among representative *Sporosarcina*, *Cellulophaga*, and *Nesterenkonia*. The trees show the relationship between the sponge-associated bacterial isolated from the Antarctic sponge *Iophon* sp. and close taxa for *Sporosarcina* spp. **(A),** *Cellulophaga* spp. **(A),** and *Nesterenkonia* spp. **(C).** Bootstrap values (1000 replications) are showed as percentage at each node. The grey boxes highlight the strains from this study.

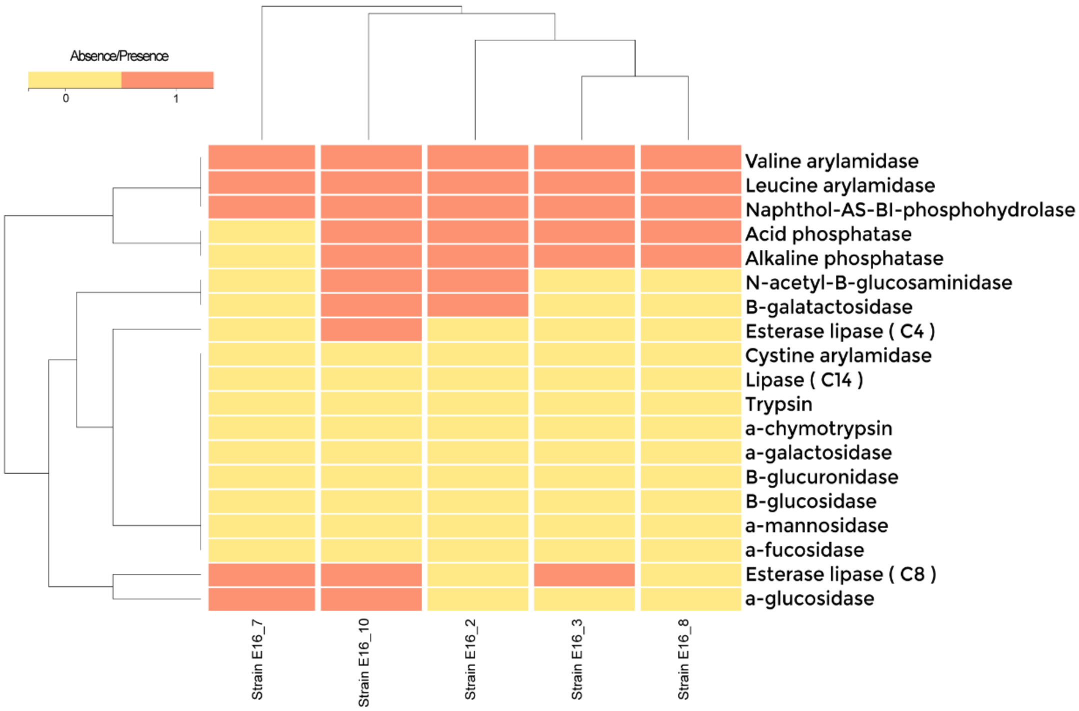

Supplementary Figure 4. Heat map of enzymatic characterization of sponge-associated *Sporosarcina*, *Cellulophaga*, and *Nesterenkonia*. The Absence and presence of enzymatic activity is represented.

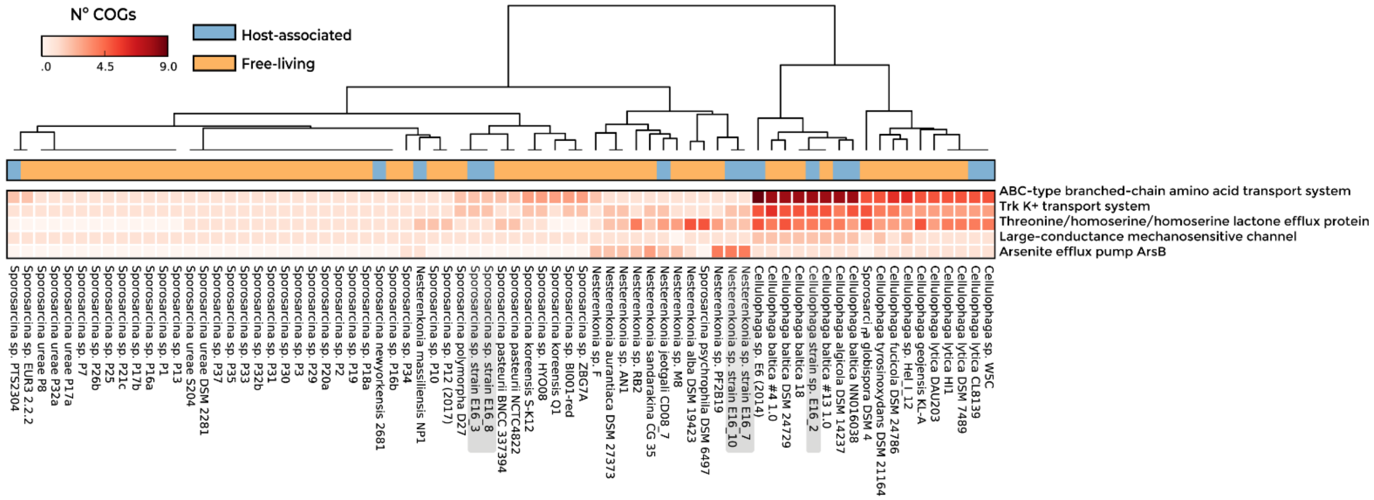

Supplementary Figure 5. Abundance of COGs involved in transporters detected in the host-associated and free-living microorganisms. Hierarchical clustering method based in UPGMA is showed. Key represent the abundance of COGs. Blue and orange represent the host-associated and free-living genomes. The grey boxes highlight the strains from this study.

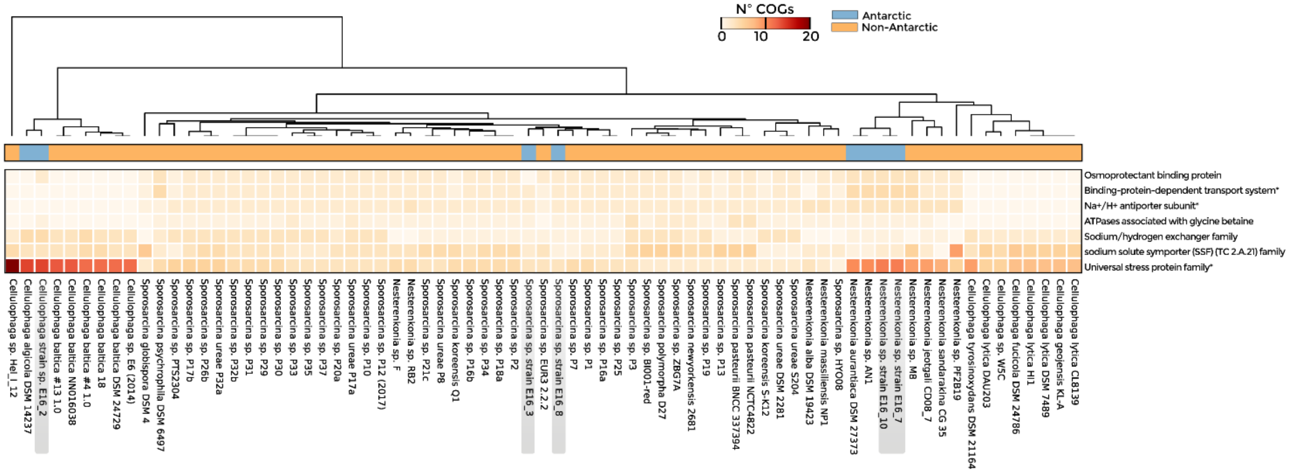

Supplementary Figure 6. Differentially abundant COGs involved in cold and osmotic tolerance detected in the host-associated and free-living microorganisms. Hierarchical clustering method based in UPGMA is showed. Color key represents the abundance of COGs. Blue and orange represent the host-Antarctic and non- Antarctic genomes. The grey boxes highlight the strains from this study.

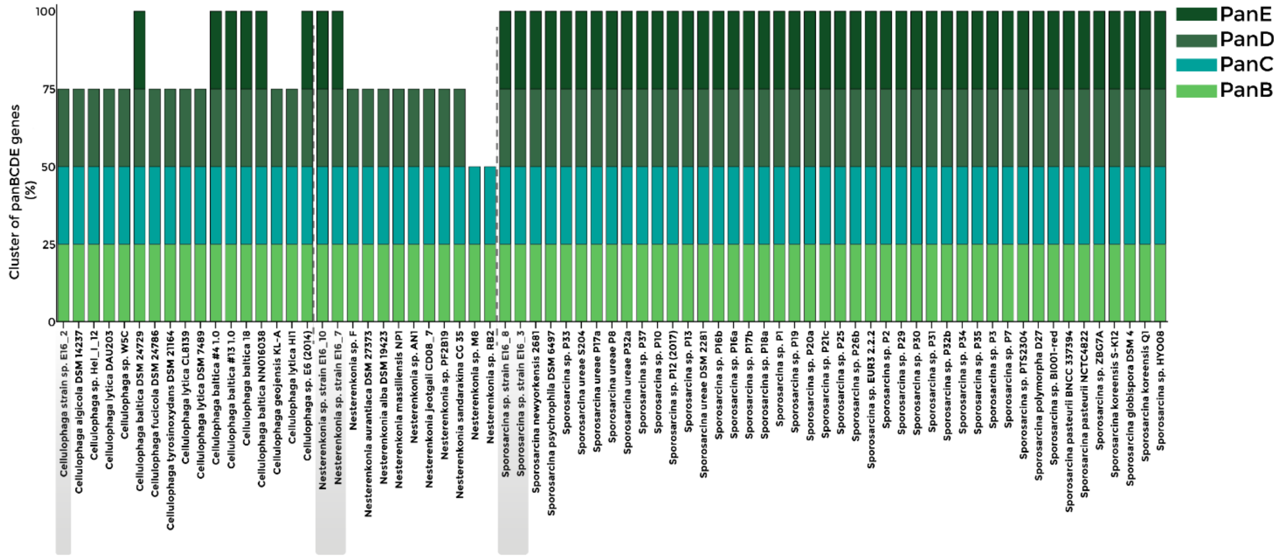

Supplementary Figure 7. Distribution of *pan*BCDE genes in the genome of *Sporosarcina*, *Cellulophaga*, and *Nesterenkonia*. The grey boxes highlight the strains from this study.

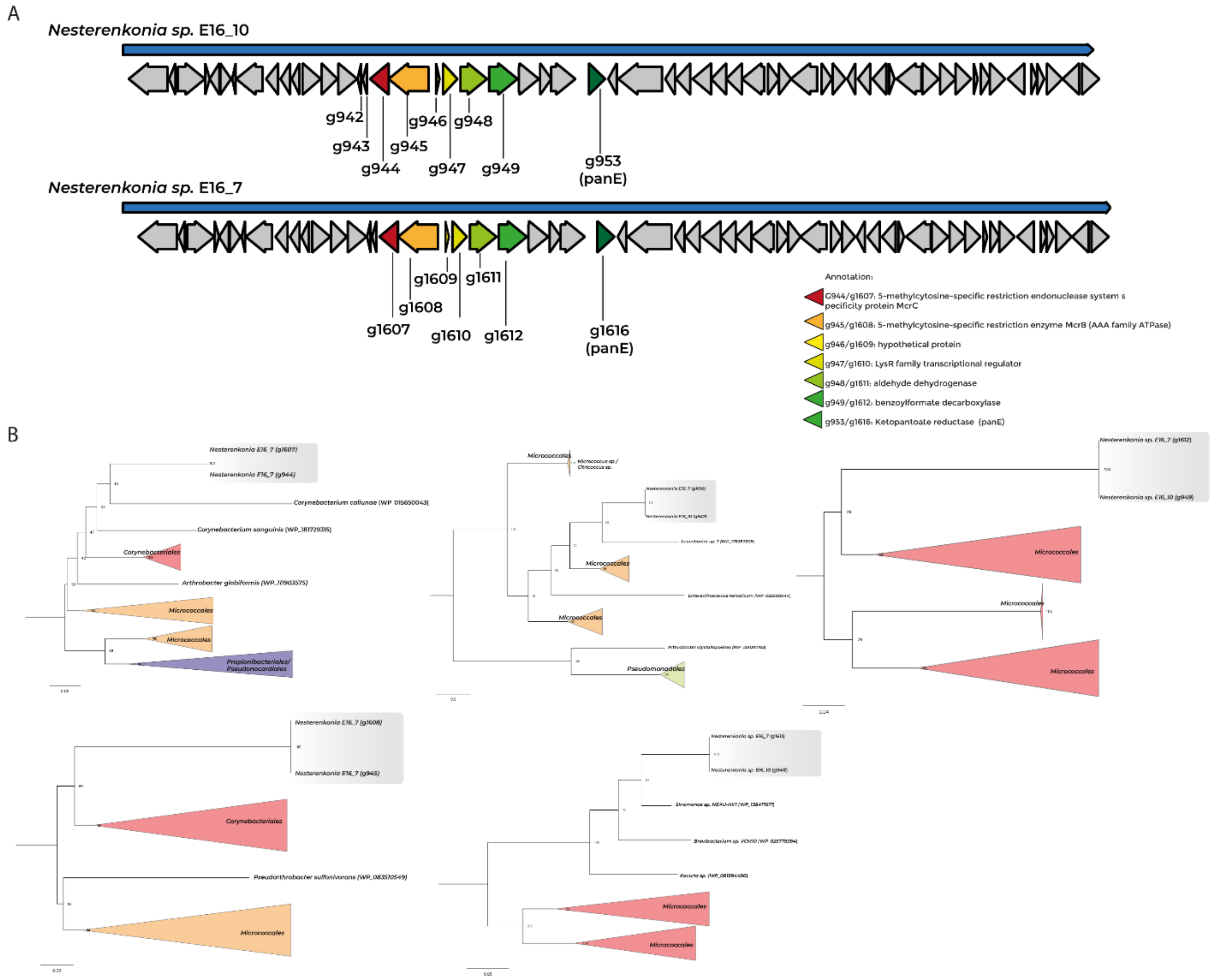

Supplementary Figure 8. Phylogenetic and synteny analyses of genes contained in the genomic island in sponge-associated *Nesterenkonia.* Synteny analysis between sponge-associated *Nesterenkonia* is shown in (A). Phylogenetic of adjacent genes to Ketopantoate reductase gene is showed in (B). The grey boxes highlight the strains from this study.
